# L-Serine Treatment May Improve Neurorestoration of Rats after Permanent Focal Cerebral Ischemia Potentially Through Improvement of Neurorepair

**DOI:** 10.1371/journal.pone.0093405

**Published:** 2014-03-26

**Authors:** Li Sun, Ren Qiang, Yao Yang, Zheng-Lin Jiang, Guo-Hua Wang, Guang-Wei Zhao, Tao-Jie Ren, Rui Jiang, Li-Hua Xu

**Affiliations:** 1 Department of Neuropharmacology, Institute of Nautical Medicine, Nantong University, Jiangsu, China; 2 Department of Infectious Diseases, The Third People's Hospital of Nantong, Jiangsu, China; 3 Department of Neurology, Affiliated Hospital, Nantong University, Jiangsu, China; St Michael's Hospital, University of Toronto, Canada

## Abstract

The present study was conducted to clarify whether treatment with L-serine can improve the brain repair and neurorestoration of rats after permanent middle cerebral artery occlusion (pMCAO). After pMCAO, the neurological functions, brain lesion volume, and cortical injury were determined. GDNF, NGF, NCAM L1, tenascin-C, and Nogo-A levels were measured. Proliferation and differentiation of the neural stem cells (NSCs) and proliferation of the microvessels in the ischemic boundary zone of the cortex were evaluated. Treatment with L-serine (168 mg/kg body weight, i.p.) began 3 h after pMCAO and was repeated every 12 h for 7 days or until the end of the experiment. L-Serine treatment: 1) reduced the lesion volume and neuronal loss; 2) improved the recovery of neurological functions; 3) elevated the expression of nerve growth-related factors; and 4) facilitated the proliferation of endogenous NSCs and microvessels activated after pMCAO and increased the number of new-born neurons. 5) D-cycloserine, an inhibitor of serine hydroxymethyltransferase, blunted the effects of L-serine on NSC proliferation, differentiation, microvascular proliferation. In conclusions, L-serine treatment in pMCAO rats can reduce brain injury and facilitate neurorestoration which is partly associated with the improvement of proliferation of NSCs and microvessels, reconstruction of neurovascular units and resultant neurorepair. The effects of L-serine on endogenous NSC proliferation and microvascular proliferation are partly mediated by the action of L-serine as a substrate for the production of one-carbon groups used for purine and pyrimidine synthesis and modulation of the expression of some nerve growth-related factors.

## Introduction

Stroke is one of the leading causes of death and disability worldwide. The pathophysiology of stroke is complicated and involves excitotoxic mechanisms, oxidative and nitrative stresses, inflammatory responses, ionic imbalances, apoptosis, angiogenesis, neuroprotection, and neurorestoration [1]–[4]. Previously developed treatment strategies for ischemic stroke primarily focused on reducing the size of ischemic damage and rescuing dying cells early after occurrence [4], [5]. A large number of neuroprotective agents have been designed to interrupt the ischemic cascade, but clinical therapeutic trials of these agents have not demonstrated consistent benefits, despite successful preceding animal studies [4], [5]. Intravenous recombinant tissue plasminogen activator is the only therapy for acute ischemic stroke approved by the United States Food and Drug Administration, but its use is limited by a narrow therapeutic window [1], [4], [5].

Alternatively, many investigators have attempted to promote neural regeneration and brain repair after injury [6]–[9] because regeneration of the brain after damage is still active days and even weeks after a stroke occurs, which might provide a second window for treatment [10]. Neuroregeneration is currently a hot subject, but there are many factors that influence the regeneration of brain tissue, and the involved mechanisms are very complicated; thus far, there has been no substantial breakthrough [6], [7]. Transplantation of cells or tissues tested to promote neural regeneration and brain tissue repair after injury includes peripheral nerve grafts, Schwann cells, embryonic brain and spinal cord tissue, olfactory ensheathing cells, embryonic stem cells, neural stem cells (NSCs), bone marrow stromal cells, activated macrophages, and others [7], [11]–[13], but the involved methodology is still in its infancy, and the efficacy of transplantation is unreliable [9], [14], [15]. An alternative method is to promote endogenous NSC proliferation [16], [17] and to help these cells migrate to the injured area, repair damaged brain tissue and restore neurological function; however, there is still a great distance to clinical application for this method [9], [16]. To achieve neurorestoration in the treatment of brain injury, at least obtaining recovery of motor and cognitive function, several things are needed including reduced brain cell loss, recruitment of existing but latent connections, and development of new neurons and neural connections [5], [18], [19].

L-Serine is a crucial neurotrophic factor and a precursor for phosphatidyl-L-serine, L-cysteine, nucleotides, sphingolipids, and neurotransmitters such as D-serine and glycine. It plays an important role in neuronal development and function in the central nervous system (CNS) [20]. Our previous studies have demonstrated that L-serine treatment at an early stage after cerebral ischemia in rats can exert a neuroprotective effect that is potentially mediated by reducing neuronal excitability through activating glycine receptors [21] and improving the cerebral blood flow through activating apamin and charybdotoxin-sensitive Ca^2+^-activated K^+^ channels on the endothelium [22]. Moreover, L-serine, as the predominant source of one-carbon groups for the *de novo* synthesis of purine nucleotides and deoxythymidine monophosphate, as reviewed by de Koning et al., plays a central role in cellular proliferation [23]. We hypothesised that treatment with L-serine after brain injury might thus facilitate neuroregeneration. Therefore, the present study was performed to clarify whether treatment with L-serine could improve the brain repair and neurorestoration of the rats after permanent focal cerebral ischemia.

## Materials and Methods

### Animals and ethics statements

Male Sprague-Dawley rats weighing 220–250 g were used in the present study (obtained from the Experimental Animal Centre of Nantong University, Nantong, China). About 10–12 rats were randomly allocated to each experimental group. Due to anesthetic accident and anesthetic side effects such as adynamic ileus, some rats died during the experiments and a part of samples were lost in each group. L-serine treatment did not significantly influence the mortality rate of rats. Behavioural measurements including neurological severity scores and learning and memory and cell counting after Nissl staining and immunochemistry were performed by investigators blinded to the treatment groups. All procedures were in strict accordance with the institutional guidelines of Nantong University, which complies with international rules and policies. Ethics in accordance with the ARRIVE (Animal Research: Reporting *In Vivo* Experiments) guidelines were followed in the animal experiments and approved by the Animal Care and Use Committee of Nantong University, Nantong, China (Permit Number: 20120210-02). All surgery was performed under anesthesia, and all efforts were made to minimize suffering.

### Chemicals

L-Serine, 2,3,5-triphenyltetrazolium chloride (TTC), 5-bromo-2′-deoxyuridine (B5002, BrdU), D-cycloserine (C3909), mouse monoclonal anti-BrdU antibody (B2513), rabbit anti-nestin antibody (N5413), fluorescein (FITC)-conjugated goat anti-rabbit IgG (whole molecule) (F9887) were purchased from Sigma-Aldrich Corporation (Saint Louis, USA). Rabbit anti-neuron-specific nuclear protein (NeuN) antibody (clone A60) from Millipore (Temecula, USA); rabbit anti-von Willebrand factor (vWF) antibody (ab6994) from Abcam (Hongkong, China); FITC-conjugated affinipure donkey anti-mouse IgG (83441) from Jackson ImmunoResearch Laboratories, Inc. (West Grove, USA); enzyme-linked immunosorbent assay kits for measurements of glia-derived neurotrophic factor (GDNF), nerve growth factor (NGF), neural cell adhesion molecule L1 (NCAM L1), tenascin-C (TN-C), and neurite outgrowth inhibitor-A (Nogo-A) from Shanghai Jijin Chemistry and Technology Co. Ltd. (Shanghai, China); and a bicinchoninic acid protein assay kit from Beyotime Institute of Biotechnology (Haimen, China) were also used. All other chemicals were from Sinopharm Chemical Reagent Co. Ltd. (Shanghai, China) or Xilong Chemical Co. Ltd. (Guangzhou, China).

### Middle cerebral artery occlusion (MCAO) surgery

Rats were fasted overnight with free access to water. All animals were anesthetised first with 2 mL of enflurane, and anesthetisation was maintained with 10% chloral hydrate (350 mg/kg body weight, i.p.). Permanent MCAO (pMCAO) was induced as reported previously [22], [24]. Briefly, after a midline neck incision, the right common carotid artery (CCA), internal carotid artery (ICA) and external carotid artery (ECA) were exposed, and the proximal ECA and CCA were then ligated. The vagus nerve was carefully preserved as far as possible. A specialised nylon suture with a 0.18 mm diameter (Beijing Sunbio Biotech Co. Ltd., Beijing, China) was used. The tip of this suture was coated with silicon gel and the diameter of it was 0.24 mm. The suture was introduced from the lumen of the distal CCA just before bifurcation into the ICA until resistance was felt. Thus, the origin of the middle cerebral artery (MCA) was occluded by the nylon suture. The average depth of filament insertion was 18.5±0.5 mm away from the bifurcation. Then, the exposed vessels were carefully ligated to prevent bleeding, and the incision was closed aseptically. Sham-operated animals were subjected to the same surgical procedure, but the suture was not advanced beyond the internal carotid bifurcation. A laser Doppler perfusion monitor (PeriFlux System 5010, Perimed, Stockholm, Sweden) was used to monitor cerebral blood flow (CBF) throughout the operation. The ischemic model was considered successful if an approximately 75% reduction in CBF was induced immediately after placement of the suture [22], [25]. Otherwise, the animals were excluded. The rectal temperature of the rats was maintained at 37±0.5°C using a temperature-regulated heating pad throughout the anaesthetic period including surgical preparation. After revival from anaesthesia, the animals were placed back into cages with the room temperature maintained at 25±2°C. Some of the rats died during the experiment, and the mortality during the first 24 h after pMCAO operation was 20.6% and 19.7%, and the additional mortality until end-point was 7.5% and 7.2%, respectively, in the vehicle and L-serine treated groups. In addition, about 30% of the rats we used in this study developed adynamic ileus with a swollen abdomen, lethargy, anorexia and resultant death several days after MCAO. Data from these animals were excluded.

### Neurological severity scores (NSS)

Neurological functions were evaluated at 1, 3, 5, 7, 10, 14, 21, 28, and 35 days after pMCAO and scored on a scale of 0 to 18 points (normal score, 0 point; maximal deficit score, 18 points) through motor, sensory, balance, and reflex tests [26], [27]. For the injury severity scores, 1 point was awarded for the inability to perform the test or for the lack of a tested reflex; thus, the higher score is, the more severe is the injury.

Motor tests (6 points) were as scored as follows: 1) raising rat by the tail (3 points)-1, flexion of forelimb; 1, flexion of hind limb; and 1, head moved >10° to vertical axis within 30 s and 2) placing rat on the floor (normal = 0 point, maximum = 3 points); 0 normal walk; 1, inability to walk straight; 2, circling toward the paretic side; and 3, fall down to the paretic side.

Sensory tests (2 points) were scored as follows: 1) placing test (visual and tactile test) and 2) proprioceptive test (deep sensation, pushing the paw against the table edge to stimulate limb muscles).

Beam balance tests (normal = 0 point, maximum = 6 points) were as scored as follows: 0, balances with steady posture; 1, grasps side of beam; 2, hugs the beam and one limb falls down from the beam; 3, hugs the beam and two limbs fall down from the beam, or spins on beam (>60 s); 4, attempts to balance on the beam but falls off (>40 s); 5, attempts to balance on the beam but falls off (>20 s); and 6, falls off, no attempt to balance or hang on to the beam (<20 s).

Reflexes absent and abnormal movements (4 points) tests were scored as follows: 1, pinna reflex (head shake when touching the auditory meatus); 1, corneal reflex (eye blink when lightly touching the cornea with cotton); 1, startle reflex (motor response to a brief noise from snapping a clipboard paper; and 1, seizures, myoclonus, and myodystony.

### Testing of behaviour in a Morris water maze

On day 6 to day 2 before the operation of pMCAO, rats were placed for 30 seconds at first on a platform (12 cm in diameter) that was hidden 2 cm below the water surface in a pool 1.6 m in diameter filled with 25°C water and then were placed in one of the three quadrants without the platform, and the time to find the platform (up to 90 seconds) was recorded. This training was repeated four times a day, and the rats were trained for consecutive 5 days. On day 1 before pMCAO, the platform was moved away from the Morris water maze (RD1101-MWM-HG, Mobiledatum Co. Ltd, Shanghai, China), the rats were placed in one of the four quadrants, and the swimming trace and the time to enter the place of platform were recorded. After pMCAO, the training was repeated on days 8–12 and on days 29–33, and the recording of swimming trace and the time to enter the place of platform was repeated again on days 13 and 34.

### Measurement of CBF and other physiological parameters

According to the literature [22], [25], CBF was continuously monitored before and during the operation of pMCAO with a laser Doppler perfusion monitor (PeriFlux System 5000, Perimed). Briefly, a sagittal skin incision approximately 1.5 cm long was made. A scanning probe (407-1, Perimed) was carefully placed on the skull ipsilateral to the pMCAO under a stereotaxic device (51653, Stoelting Co., Wood Dale, IL, USA) with its centre at 5 mm lateral to midline and 1 mm posterior to bregma, thus avoiding any large vessel. During the measurement of CBF, transcutaneous partial pressure of O_2_ (tcPO_2_) was simultaneously monitored from the hip skin of the rat using a probe (E5250, Perimed) [28]. Blood pressure and heart rate were simultaneously measured with a caudal artery pressure measuring system (Alcott Biotech Co. Ltd., Shanghai, China).

### Assessment of lesion volume

Cerebral lesion size was assessed using the TTC staining method [29]. Animals were anesthetised with 10% chloral hydrate (350 mg/kg, i.p.) at 35 days after pMCAO, and their brains were removed and sectioned into consecutive 2 mm-thick coronal slices using a mould (RBM-4000C, ASI Instruments, Warren, MI, USA). The slices were immediately immersed into 1% TTC medium at 37°C for 15 min and then turned over for another 15 min. The stained slices were washed in phosphate-buffered saline (PBS) for 5 min and then fixed in 4% buffered formaldehyde solution for 24 h. At the end of staining and fixation, colour images of these slices were captured using a video camera (PowerShot S60, Canon, Tokyo, Japan). The lesion volume was analysed using Image Pro Plus software. The percentage lesion volume was calculated as follows: [(V_C_–V_L_)/2V_C_]×100, where V_C_ is the volume of control hemisphere (left side) and V_L_ the volume of non-injured tissue in the injured hemisphere (right side).

### Nissl staining

Thirty-five days after pMCAO, rats were anaesthetised with 10% chloral hydrate (350 mg/kg, i.p.) and perfused with 250 mL of normal saline and subsequently with 200 mL of 4% paraformaldehyde in 0.1 M PBS (pH 7.4). The rat brains were then removed and post-fixed for 24 h in the same fixative. The post-fixed brain tissues were cryo-protected in 20% then 30% sucrose in PBS. The brain tissues were then sectioned coronally with 20 μm thickness with a cryostat slicer (CM1900, Leica, Bensheim, Germany). The sections from bregma -2.92 mm to -3.72 mm were prepared, mounted with neutral balata (Shanghai Specimen and Model Factory, Shanghai, China) and blotted onto slides, and then covered with a coverslip after Nissl staining, which was performed as reported previously [21].

After Nissl staining, neuronal cells in the cortex along the lesion area were identified under a high-magnification (×400) light microscope and counted by an investigator blinded to the grouping. For each rat, the mean number of neurons was obtained by examining three serial coronal sections. In each section, the number of neurons was averaged from three different vision fields of the cortex. Only intact neurons with a clearly defined cell body and nucleus were counted.

### Enzyme-linked immunosorbent assay (ELISA)

The concentrations of GDNF, NGF, NCAM L1, TN-C, and Nogo-A in the brain tissue of both sides were measured with ELISA in three groups of rats: the sham-operated, vehicle, and L-serine-treated groups (each n = 6). Five days after pMCAO, each rat brain was harvested under anaesthesia of 10% chloral hydrate (350 mg/kg, i.p.), frozen and stored at −80°C until use. Brain homogenates were centrifuged at 4°C and 14,000 rpm for 30 min. Supernatants were transferred to new Eppendorf tubes, and the protein concentration of the supernatants was measured with a bicinchoninic acid protein assay kit. Then, the supernatants were assayed in duplicate using GDNF, NGF, NCAM L1, TN-C, and Nogo-A assay kits according to the manufacturer's recommendations.

### Immunohistochemistry

To examine the effects of L-serine on the proliferation and differentiation of NSCs and the proliferation of vascular endothelial cells, the rats were sacrificed under anaesthesia of 10% chloral hydrate (350 mg/kg, i.p.) by transcardial perfusion with PBS on day 6 for detecting BrdU/nestin-positive NSCs and vWF-positive endothelial cells and on day 14 for detecting BrdU/NeuN-positive neurons after pMCAO, and the brains were removed and post-fixed for 24 h in 4% paraformaldehyde. The post-fixed brains were cryo-protected in PBS containing 25% sucrose. The brains were then sectioned to 5-μm thickness with a cryostat slicer (CM1900, Leica, Bensheim, Germany). BrdU was dissolved in PBS (6.25 mg/mL) and injected into rats (50 mg/kg body weight, i.p.) twice daily from day 3 to day 5 after pMCAO.

For immunofluorescent staining, the brain sections were washed three times with 0.01 mol/L PBS containing 0.3% Triton X (wash buffer, pH 7.4) for 30 min, incubated with 2 N HCl for 20 min at 37°C, washed three times again with wash buffer for 30 min, and blocked with 3% normal donkey or goat serum (Jackson, West Grove, USA) in wash buffer for 60 min at room temperature. The sections were then incubated in blocking solution with primary antibody, mouse anti-BrdU antibody (1∶500), rabbit anti-nestin antibody (1∶150), rabbit anti-NeuN antibody (1∶400), or rabbit anti-vWF antibody (1∶400) at 4°C overnight and subsequently with secondary antibody FITC-conjugated Affinipure donkey anti-mouse IgG (1∶500) or FITC-conjugated goat anti-rabbit IgG (1∶1000) at room temperature for 2 h. The sections were covered with coverslips for microscope observation after antibody incubation. Fluorescent microscopic images were obtained with a computer-driven microscope (Leica DMLB, Solms, Germany) and using Leica Qwin V3 software (Leica Corporation). The immunopositive cells were counted in three non-overlapping fields under a high magnification (×200) fluorescent microscope by an observer blinded to the individual treatments. The number of positively labelled cells in the ischemic boundary zone (IBZ, adjacent to the ischemic core area) of the cortex was averaged in three random sections taken from each animal.

### L-serine administration

Rats were randomly divided into three groups except those specified elsewhere: sham-operated group, vehicle group, and L-serine treated group. L-serine was dissolved in normal saline, and treatment with L-serine (168 mg/kg body weight, i.p.) or vehicle was started at 3 h after pMCAO and repeated every 12 h for 7 days or until the end of the experiment. D-cycloserine was used to inhibit serine hydroxymethyltransferase [30], [31]. D-cycloserine was dissolved in normal saline and injected intraperitoneally at 5 mg/kg and 15 mg/kg body weight just before each use of L-serine.

### Statistics

The data are presented as the mean ± standard error. The neurological severity score data were compared using the Kruskal-Wallis rank-sum test and the extended t-test for post hoc comparisons. The data from multiple groups were analysed using one-way ANOVA and the Newman Keuls test for post hoc comparisons. Other data of the two groups were analysed using Student's t-test. Differences with *p* values less than 0.05 were considered statistically significant.

## Results

### Monitoring of the physiological parameters

Blood pressure, heart rate and tcPO_2_ were monitored; however, no notable influence was observed after the administration of L-serine, D-cycloserine, or a combination of L-serine and D-cycloserine (data no shown).

### L-Serine treatment reduced the lesion volume and neuronal loss

**Figure 1:**
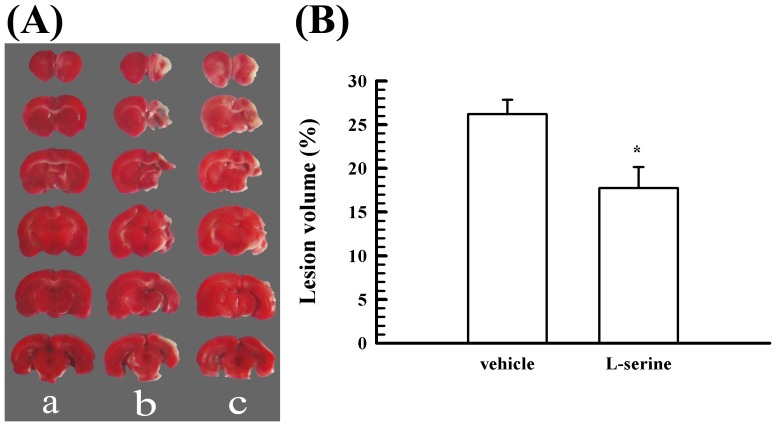
Effect of L-serine treatment on the lesion volume 35 days after pMCAO. (A) Representative examples of TTC staining after different treatments: a, sham-operated group; b, control group; c, L-serine (168 mg/kg) treated group. (B) Mean values of the lesion volume after different treatments (n = 5). ^*^ *p*<0.05 *vs*. vehicle group.

Thirty-five days after pMCAO, the injured brain tissue became liquefied and was easily lost after being sectioned because of liquefaction, but measurement of the lesion volume was not influenced (Fig. 1A). L-serine treatment notably reduced the lesion volume, and the brain tissue loss during sectioning was less in the L-serine-treated group than in the vehicle group (*p*<0.05, Fig. 1A and B).

**Figure 2:**
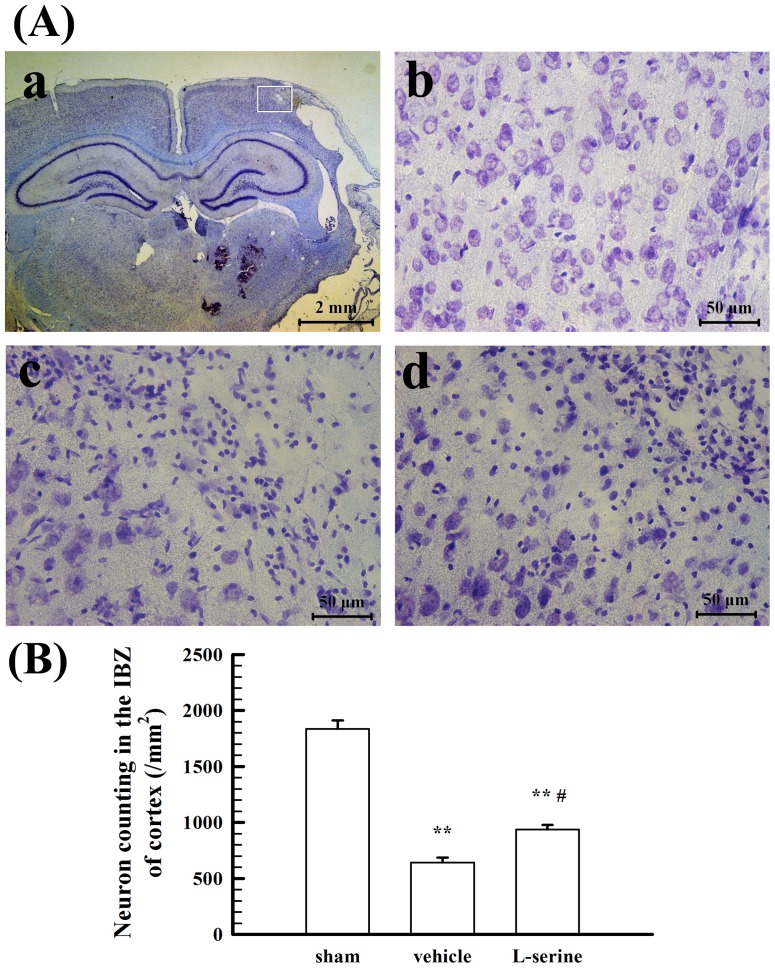
Nissl staining and cell counting of the IBZ of rat cortices 35 days after pMCAO. (A) Representative examples of Nissl staining: a, quadrate box is the area of neurons counted; b, sham-operated group; c, vehicle group; d, L-serine (168 mg/kg) treated group. (B) Mean values of neurons counted for each group (n = 5). ^**^ *p*<0.01 *vs*. sham-operated group; ^#^ *p*<0.05 *vs*. vehicle group.

Moreover, neuronal loss was evident at the core of injured cortex, but many residual neurons were found in the IBZ (Fig. 2A-c and B), and more residual neurons were found in the L-serine-treated group than in the vehicle group (*p*<0.05, Fig. 2A-d and B).

### L-Serine treatment improved the recovery of neurological functions

**Figure 3:**
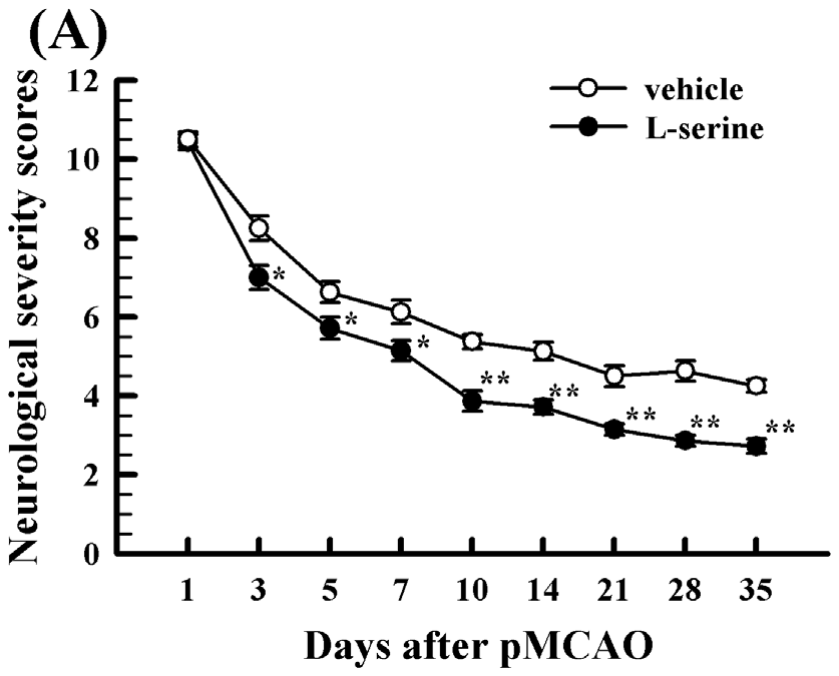
Effect of L-serine treatment on the behavioural tests in rats after pMCAO (n = 6). Neurological severity scores were measured from day 1 to day 35 after pMCAO.

Except for those in the sham-operated group, the rats in other two groups displayed severe neurological deficits after pMCAO, neurological severity scores were increased prominently at 24 h after pMCAO surgery (Fig. 3) and decreased gradually from day 3 to day 35 after pMCAO (*p*<0.05 or 0.01, Fig. 3). Compared with vehicle rats, however, neurological severity scores were reduced significantly by L-serine treatment (Fig. 3). On day 35 after pMCAO, the mean value of NSS was 2.71±0.18 in the L-serine treated group, being lower than that of 4.25±0.16 in the vehicle group (*p*<0.01, Fig. 3).

**Figure 4:**
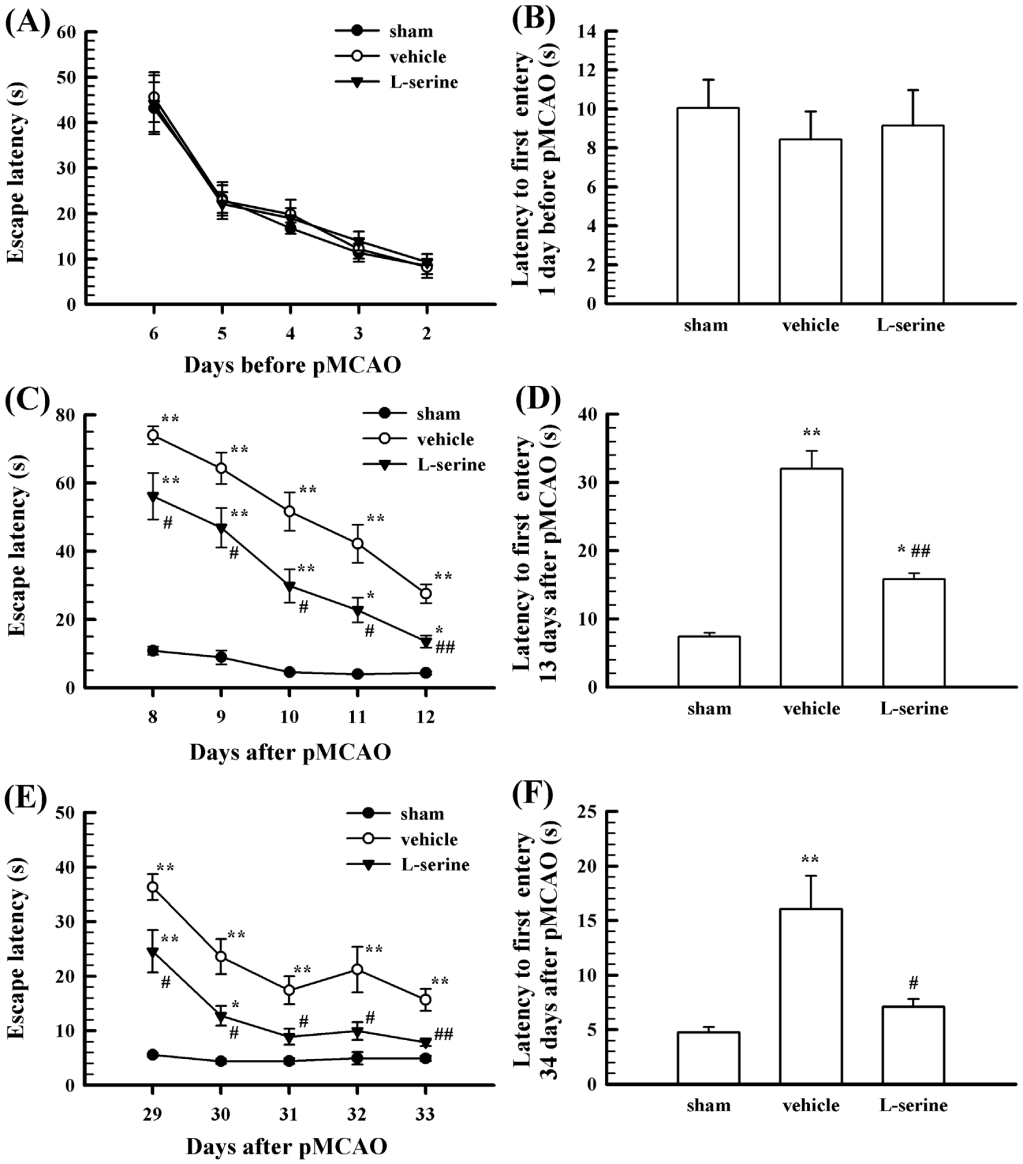
Effect of L-serine treatment on the learning and memory in rats after pMCAO (n = 6–8). (A), (C), and (E): Escape latency to find the hidden platform during five consecutive days of training before or after pMCAO. (B), (D), and (F): Latency of the first time to enter the former platform location on the test day before or after pMCAO. ^*^ *p*<0.05, ^**^ *p*<0.01 *vs*. sham-operated group; ^#^ *p*<0.05, ^##^ *p*<0.01, *vs*. vehicle group.

In addition, the learning and memory abilities of rats were measured before and after pMCAO. Before pMCAO, the learning and memory abilities did not display any significant differences in all three groups (Fig. 4A and B). After pMCAO, the time to find the platform during the training days (day 8 to day 12) was prolonged remarkably, and the time to enter the platform location on the test day (day 13) was also prolonged compared with the sham-operated group (*p*<0.01, Fig. 4C and D) or compared with the pre-pMCAO values in the vehicle group, suggesting that the learning and memory abilities of the rats were reduced after pMCAO. However, L-serine treatment significantly decreased the time to find the platform during the training days and the time to enter the platform location on the test day (*p*<0.05 or 0.01, Fig. 4C and D). Similarly, the time to find the platform on day 29 to day 33 (the training days) and the time to enter the platform location on the test day (day 34) were both shortened by L-serine treatment (*p*<0.05 or 0.01, Fig. 4E and F), suggesting that L-serine treatment could improve the learning and memory abilities of the rats after pMCAO.

### L-Serine treatment elevated the expression of nerve growth-related factors in the brain tissue

**Figure 5:**
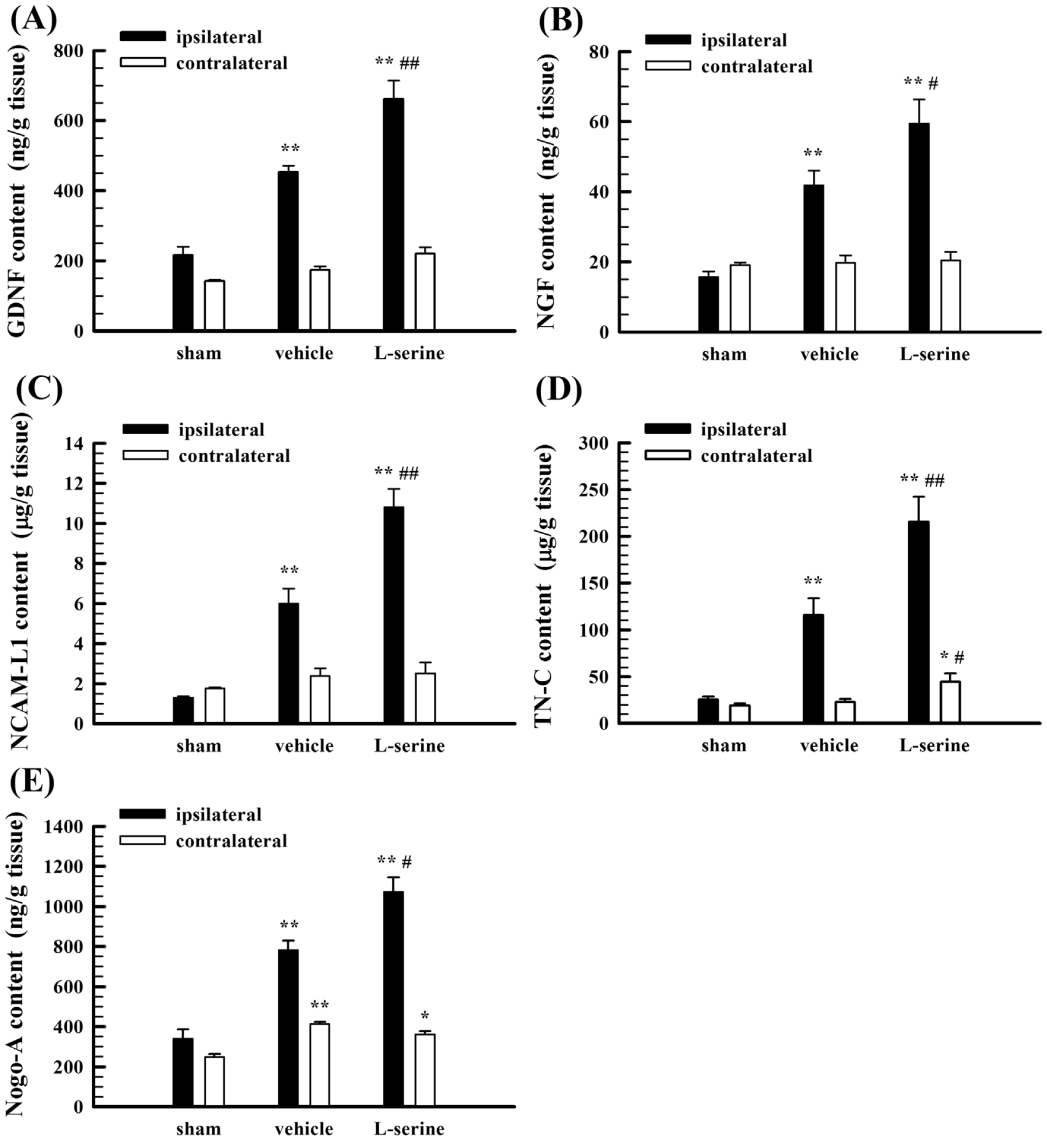
Effect of L-serine treatment on the expression of nerve growth-related factors in both sides of the brain tissue in rats 5 days after pMCAO (n = 6). (A) Changes in the expression of GDNF in different groups. (B) Changes in the expression of NGF. (C) Changes in the expression of NCAM L1. (D) Changes in the expression of TN-C. (E) Changes in the expression of Nogo-A. ^*^ *p*<0.05, ^**^ *p*<0.01 *vs*. sham-operated group; ^#^ *p*<0.05, ^##^ *p*<0.01 *vs*. vehicle group.

Compared to the sham-operated group, the expression of GDNF, NGF, NCAM L1, TN-C, and Nogo-A in the brain tissue ipsilateral to the injured side in the vehicle group was increased after pMCAO (*p*<0.01, Fig. 5), and L-serine treatment further elevated the expression of GDNF, NGF, NCAM L1, TN-C, and Nogo-A in the brain of the injured side (*p*<0.05 or 0.01, Fig. 5). However, the expression of GDNF, NGF, and NCAM L1 in the brain tissue contralateral to the injured side was not significantly changed (Fig. 5).

### L-Serine treatment increased the proliferation of NSCs and differentiation into neurons in the IBZ of cortex

**Figure 6:**
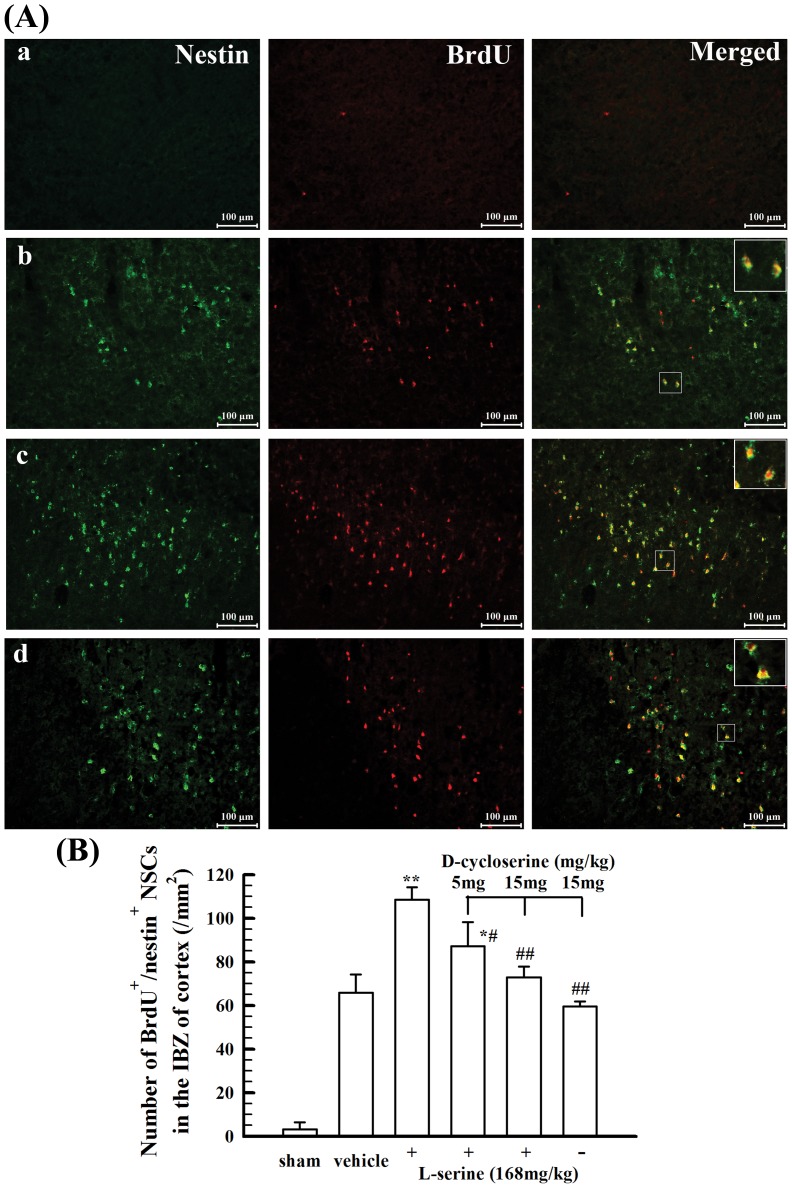
Effect of L-serine treatment on endogenous NSC proliferation in the IBZ of cortex 6 days after pMCAO. (A) Representative examples of BrdU- and nestin-positive cells: a, sham-operated group; b, vehicle group; c, L-serine (168 mg/kg) treated group; d, L-serine (168 mg/kg) and D-cycloserine (15 mg/kg) treated group. (B) Mean values of the number of BrdU^+^/nestin^+^ NSCs in the IBZ of cortex (n = 4–6). ^*^ *p*<0.05, ^**^ *p*<0.01 *vs.* vehicle group; ^#^ *p*<0.05, ^##^ *p*<0.01 *vs.* L-serine-treated group.

To observe the proliferation of NSCs, BrdU^+^/nestin^+^ cells were counted in the IBZ of cortex 5 days after pMCAO. As shown in Fig. 6, ischemic insult induced an increase of BrdU^+^/nestin^+^ cells, and L-serine treatment further increased the number of BrdU^+^/nestin^+^ cells in the IBZ of cortex 5 days after pMCAO (*p*<0.01, Fig. 6), suggesting that the ischemic insult could activate the proliferation of endogenous NSCs, and L-serine treatment can facilitate this activated proliferation of endogenous NSCs.

To verify the mechanism underlying this facilitating effect of L-serine on the proliferation of endogenous NSCs, D-cycloserine was used to inhibit serine hydroxymethyltransferase (SHMT). The pathway of L-serine catabolism initiated by SHMT is the major source of one-carbon groups, providing formyl groups for purine synthesis and methyl groups for pyrimidine synthesis, the remethylation of homocysteine and many other methylation reactions [23]. As a result, co-application of D-cycloserine blunted the effect of L-serine in a dose-dependent manner, whereas use of D-cycloserine alone did not influence the number of BrdU^+^/nestin^+^ NSCs (Fig. 6), suggesting that conversion of L-serine into one-carbon groups through the SHMT-initiated L-serine catabolism pathway may be potentially involved in the facilitating effect of L-serine on the proliferation of endogenous NSCs.

**Figure 7:**
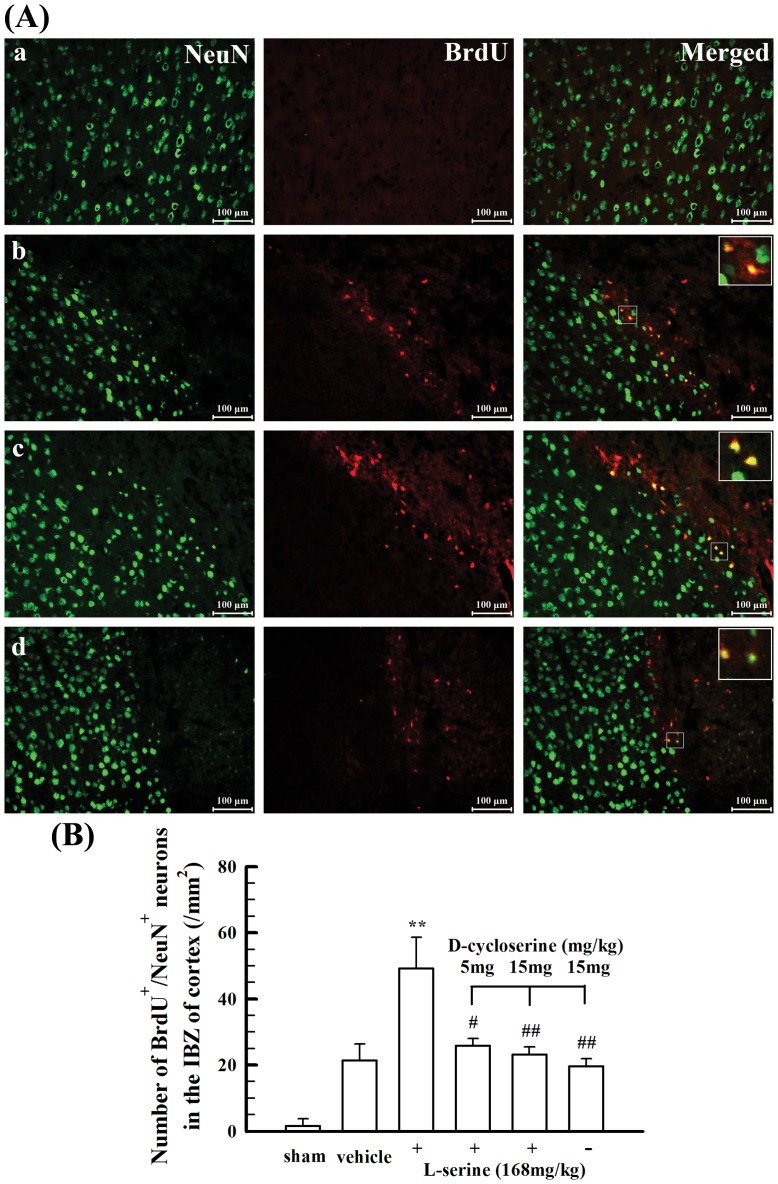
Effect of L-serine treatment on the new-born neurons in the IBZ of cortex 14 days after pMCAO. (A) Representative examples of BrdU- and NeuN-positive cells: a, sham-operated group; b, vehicle group; c, L-serine (168 mg/kg) treated group; d, L-serine (168 mg/kg) and D-cycloserine (15 mg/kg) treated group. (B) Mean values of the number of BrdU^+^/NeuN^+^ neurons in the IBZ of cortex (n = 4–6). ^**^ *p*<0.01 *vs*. vehicle group; ^#^ *p*<0.05, ^##^ *p*<0.01 *vs*. L-serine-treated group.

Moreover, we observed the mature neurons differentiating from NSCs in the IBZ of cortex and the influence of L-serine treatment in rats 14 days after pMCAO. As shown in Fig. 7, BrdU^+^/NeuN^+^ neurons were found in the IBZ of cortex after pMCAO, and L-serine treatment increased the number of BrdU^+^/NeuN^+^ neurons (*p*<0.01, Fig. 7). However, co-application of D-cycloserine inhibited the effect of L-serine, whereas D-cycloserine alone did not exert any notable influence on the number of BrdU^+^/NeuN^+^ neurons (Fig. 7). These results suggest that the new-born neurons differentiating from endogenous NSCs in the IBZ of cortex were increased after L-serine treatment in pMCAO rats.

### L-Serine treatment increased the proliferation of endothelial cells in the cortex

**Figure 8:**
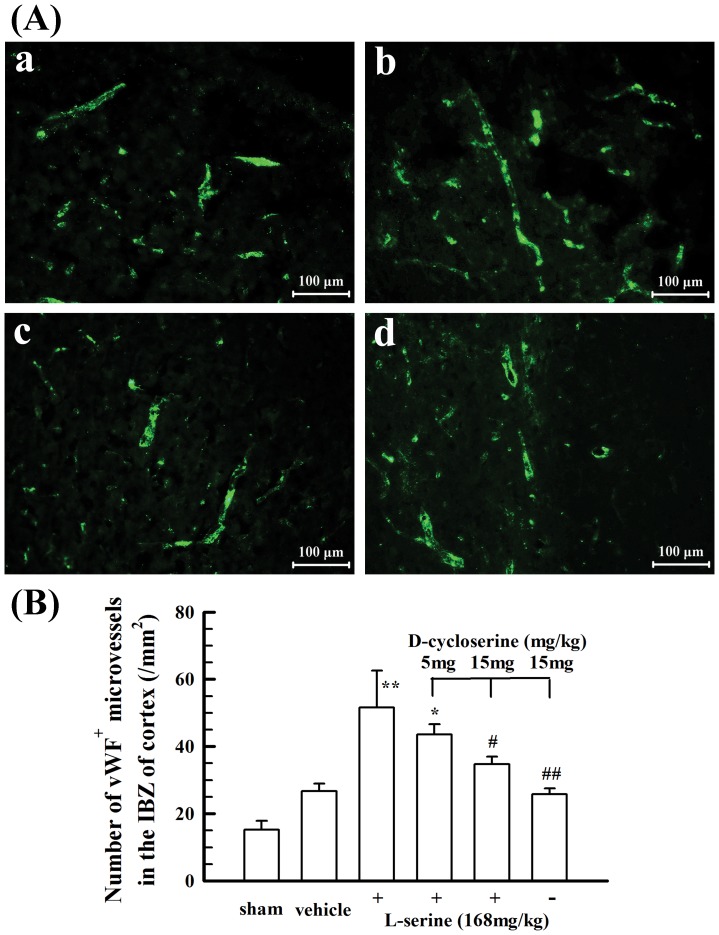
Effect of L-serine treatment on the number of microvessels in the IBZ of cortex 5 days after pMCAO. (A) Representative examples of vWF-positive microvessels: a, vehicle group; b, L-serine (168 mg/kg) treated group; c, L-serine (168 mg/kg) plus D-cycloserine (15 mg/kg) treated group; d, D-cycloserine (15 mg/kg) treated group. (B) Mean values of the microvessel number in the IBZ of cortex in different group rats (n = 4). ^*^ *p*<0.05, ^**^ *p*<0.01 *vs*. vehicle group; ^#^ *p*<0.05, ^##^ *p*<0.01 *vs*. L-serine-treated group.

To investigate the potential effect of L-serine treatment on microvascular proliferation, vWF^+^ endothelial cells were observed in the IBZ of rat cortex 5 days after pMCAO. The total number of vWF-positive microvessels was divided by the total tissue-area to determine vascular density. Data are presented as the number of vWF-positive microvessels/mm^2^. As shown in Fig. 8, L-serine treatment increased the number of vWF^+^ microvessels (*p*<0.01); however, co-application of D-cycloserine blunted the increasing effect of L-serine, whereas D-cycloserine alone did not exert any notable influence on the number of vWF^+^ microvessels (Fig. 8).

## Discussion

The present study found that L-serine treatment in rats after pMCAO reduced the lesion volume and neuronal loss, decreased neurological severity scores and improved the recovery of neurological functions, including motor, sensory, balance, reflex, learning and memory, suggesting that L-serine could reduce the brain injury in permanent cerebral ischemic rats and facilitate neurorestoration. These results are consistent with our previous observations on the therapeutic effect of L-serine in transient and permanent cerebral ischemic rats [21], [22]. The present study extended the observation time to 35 days after cerebral ischemia and more parameters, especially the learning and memory that were used to evaluate the recovery of neurological functions in rat.

To investigate the mechanisms underlying the neurorestorative effect of L-serine, we investigated the therapeutic effect of L-serine on neurorepair, including neural stem cell proliferation and differentiation and microvascular proliferation. We found that L-serine treatment facilitated the proliferation of endogenous NSCs activated after pMCAO and increased the number of new-born neurons differentiating from these NSCs in the IBZ of cortex in pMCAO rats. Moreover, L-serine treatment increased the microvascular proliferation in the IBZ of cortex. Thus, reconstruction of neurovascular units in the injured cortex after cerebral ischemia would be facilitated after treatment with L-serine. These results of L-serine treatment have not been previously reported. Furthermore, to understand the mechanisms underlying the facilitating effect of L-serine treatment on neurorepair, D-cycloserine, an inhibitor of SHMT was used. D-cycloserine blunted the effects of L-serine treatment on NSC proliferation and differentiation and microvascular proliferation. It is suggested that the facilitating effect of L-serine treatment on endogenous NSC proliferation and microvascular proliferation is potentially mediated by the action of L-serine as a substrate for the production of one-carbon groups used for purine and pyrimidine synthesis [23]. The increase in the number of new-born neurons differentiating from endogenous NSCs in the IBZ of cortex in pMCAO rats after L-serine treatment may be not the direct action of L-serine on neural differentiation and might relate to the facilitating effect of L-serine treatment on endogenous NSC proliferation because the SHMT inhibitor D-cycloserine inhibited the increasing effect of L-serine treatment on the number of new-born neurons, and as a result, more NSCs could differentiate into neurons.

In addition, the present study found that L-serine treatment elevated the expression of five nerve growth related factors: GDNF, NGF, NCAM L1, TN-C, and Nogo-A in the ischemic brain tissue of rats after pMCAO. GDNF, an important neurotrophic factor, has been demonstrated to promote the survival of many neurons, neurite outgrowth, and synaptogenesis [32], [33]. Duan et al. found that GDNF may improve the proliferation of endogenous NSCs in humans after cerebral ischemia [34]. Moreover, exogenous GDNF may exert a neuroprotective effect on the brain in different experimental models of focal and global brain ischemia [33], [35], [36]. NGF is also important for the survival, maintenance and regeneration of specific neuronal populations in the adult brain [32], [37]. Intracerebroventricular or intracerebral administration of NGF will improve neurological recovery after cerebral ischemia or traumatic brain injury [38], [39]. NCAM L1 has critical roles in axon outgrowth and fasciculation, neuronal migration and survival, and synaptic plasticity during nervous system development and promotes regeneration in animal models of acute and chronic injury of the adult nervous system [40]–[42]. In addition, some studies have indicated that transplantation of embryonic stem cells that express the NCAM L1 supported the regrowth of corticospinal axons, promoted spinal cord regeneration and improved functional recovery after spinal cord injury [43]–[45].

TN-C, a major extracellular matrix glycoprotein of the CNS, is strongly upregulated after injuries of the CNS, but its role in tissue repair is not understood. Both regeneration promoting and inhibiting roles of TN-C have been proposed, considering its abilities to both support and restrict neurite outgrowth in vitro [46], [47]. However, a recent report by Chen et al. indicates that the spontaneous recovery of locomotor functions after spinal cord injury is impaired in adult TN-C-deficient (TN-C^–/–^) mice in comparison with wild-type (TN-C^+/+^) mice. The impaired recovery was associated with reduced sprouting of monaminergic axons in the spinal cord and enhanced post-traumatic degeneration of corticospinal axons. The degeneration of corticospinal axons in TN-C^–/–^ mice was normalised to TN-C^+/+^ levels by application of the alternatively spliced TN-C fibronectin type III homologous domain D, and overexpression of this domain D via adeno-associated virus in wild-type mice improved locomotor recovery, increased monaminergic axons sprouting, and reduced lesion scar volume after spinal cord injury [46]. Moreover, some other studies indicate that TN-C supports the growth of axons with appropriate receptors, including some integrins and F3/F11/contactin [48]–[50]. The results reported by Abaskharoun et al. suggest that phosphacan/receptor protein-tyrosine phosphatase-ζ/β and its ligand, tenascin-C, expressed by neural stem cells and neurons derived from embryonic stem cells, may be important for the therapeutic application in facilitating nervous tissue repair and regeneration [51].

Therefore, increases in the expression of these four nerve growth-related factors (GDNF, NGF, NCAM L1, and TN-C) after L-serine treatment in pMCAO rats may help the brain tissue produce a microenvironment better for the proliferation, survival, and differentiation of NSCs and for the proliferation of microvessels and, as a result, promote the reconstruction of neurovascular units and the repair of the injured brain tissue and improve the neurorestoration of rats. These are novel mechanisms underlying the therapeutic effect of L-serine for the rats with ischemic brain injury, in addition to the glycine receptor-mediated neuroprotective mechanism and the endothelial apamin- and charybdotoxin-sensitive Ca^2+^-activated K^+^ channels-mediated vasodilation mechanism we reported previously [21], [22]. Thereby, combining with present results we reported here, we suggest that L-serine might be a new candidate for the treatment of ischemic brain injury because of its actions on multiple targets.

Nogo-A, the largest member of the Nogo family, generally known as a neurite outgrowth inhibitor [52], [53], is responsible for inhibition of CNS regeneration. However, a recent study by Pernet et al. demonstrated an effect of Nogo-A likely beneficial to neuroregeneration, i.e., the axonal growth in the optic nerve activated by the intraocular injection of the inflammatory molecule Pam3Cys tended to be lower in Nogo-A knock-out mice than in wild-type mice, and Nogo-A overexpression in retinal ganglion cells in vivo or in the neuronal cell line F11 in vitro promoted regeneration, demonstrating a positive, cell-autonomous role for neuronal Nogo-A in the modulation of axonal regeneration [54]. Therefore, whether the increase in Nogo-A expression after L-serine treatment in the present study is beneficial to neuroregeneration and neurorestoration needs to be clarified in a future study.

In conclusion, L-serine treatment in pMCAO rats can reduce brain injury and facilitate neurorestoration which is partly associated with the improvement of proliferation of NSCs and microvessels, reconstruction of neurovascular units and resultant neurorepair. The effects of L-serine on endogenous NSC proliferation and microvascular proliferation are partly mediated by the action of L-serine as a substrate for the production of one-carbon groups used for purine and pyrimidine synthesis and modulation of the expression of some endogenous nerve growth-related factors.

## References

[1] Moskowitz MA, Lo EH, Iadecola C (2010) The science of stroke: mechanisms in search of treatments. Neuron 67: 181–198. 20670828 10.1016/j.neuron.2010.07.002 PMC2957363

[2] Macrae IM (2011) Preclinical stroke research-advantages and disadvantages of the most common rodent models of focal ischaemia. Br J Pharmacol 164: 1062–1078. 21457227 10.1111/j.1476-5381.2011.01398.x PMC3229752

[3] Broughton BR, Reutens DC, Sobey CG (2009) Apoptotic mechanisms after cerebral ischemia. Stroke 40: e331–e339. 19182083 10.1161/STROKEAHA.108.531632

[4] Pandya RS, Mao L, Zhou H, Zhou S, Zeng J, et al (2011) Central nervous system agents for ischemic stroke: neuroprotection mechanisms. Cent Nerv Syst Agents Med Chem 11: 81–97. 21521165 10.2174/187152411796011321 PMC3146965

[5] Sahota P, Savitz SI (2011) Investigational therapies for ischemic stroke: neuroprotection and neurorecovery. Neurotherapeutics 8: 434–451. 21604061 10.1007/s13311-011-0040-6 PMC3250280

[6] Picard-Riera N, Nait-Oumesmar B, Baron-Van Evercooren A (2004) Endogenous adult neural stem cells: limits and potential to repair the injured central nervous system. J Neurosci Res 76: 223–231. 15048920 10.1002/jnr.20040

[7] Richardson RM, Singh A, Sun D, Fillmore HL, Dietrich DW, et al (2010) Stem cell biology in traumatic brain injury: effects of injury and strategies for repair. J Neurosurg 112: 1125–1138. 19499984 10.3171/2009.4.JNS081087

[8] Xiong Y, Mahmood A, Chopp M (2010) Neurorestorative treatments for traumatic brain injury. Discov Med 10: 434–442. 21122475 PMC3122155

[9] Xiong Y, Mahmood A, Chopp M (2010) Angiogenesis, neurogenesis and brain recovery of function. Curr Opin Investig Drugs 11: 298–308. PMC2836170 20178043

[10] Thored P, Arvidsson A, Cacci E, Ahlenius H, Kallur T, et al (2006) Persistent production of neurons from adult stem cells during recovery after stroke. Stem cells 24: 739–747. 16210404 10.1634/stemcells.2005-0281

[11] Chiu SC, Hung HS, Lin SZ, Chiang E, Liu DD (2009) Therapeutic potential of olfactory ensheathing cells in neurodegenerative diseases. J Mol Med (Berl) 87: 1179–1189. 19756447 10.1007/s00109-009-0528-2

[12] Shear DA, Tate CC, Tate MC, Archer DR, LaPlaca MC, et al (2011) Stem cell survival and functional outcome after traumatic brain injury is dependent on transplant timing and location. Restor Neurol Neurosci 29: 215–225. 21697596 10.3233/RNN-2011-0593

[13] Vaquero J, Zurita M (2010) Functional recovery after severe CNS trauma: current perspectives for cell therapy with bone marrow stromal cells. Prog Neurobiol 93: 341–349. 21163325 10.1016/j.pneurobio.2010.12.002

[14] Gögel S, Gubernator M, Minger SL (2011) Progress and prospects: stem cells and neurological diseases. Gene Ther 18: 1–6. 20882052 10.1038/gt.2010.130

[15] Jablonska A, Lukomska B (2011) Stroke induced brain changes: implications for stem cell transplantation. Acta Neurobiol Exp (Wars) 71: 74–85. 21499328 10.55782/ane-2011-1824

[16] Leker RR (2009) Fate and manipulations of endogenous neural stem cells following brain ischemia. Expert Opin Biol Ther 9: 1117–1125. 19653861 10.1517/14712590903130558

[17] Yoneyama M, Shiba T, Hasebe S, Ogita K (2011) Adult neurogenesis is regulated by endogenous factors produced during neurodegeneration. J Pharmacol Sci 115: 425–432. 21422724 10.1254/jphs.11r02cp

[18] Bednar MM, Perry A (2012) Neurorestoration therapeutics for neurodegenerative and psychiatric disease. Neurol Res 34: 129–142. 22333068 10.1179/1743132811Y.0000000069

[19] Seo JH, Cho SR (2012) Neurorestoration induced by mesenchymal stem cells: potential therapeutic mechanisms for clinical trials. Yonsei Med J 53: 1059–1067. 23074102 10.3349/ymj.2012.53.6.1059 PMC3481376

[20] de Koning TJ, Klomp LW (2004) Serine-deficiency syndromes. Curr Opin Neurol 17: 197–204. 15021249 10.1097/00019052-200404000-00019

[21] Wang GH, Jiang ZL, Chen ZQ, Li X, Peng LL (2010) Neuroprotective effect of L-serine against temporary cerebral ischemia in rats. J Neurosci Res 88: 2035–2045. 20186763 10.1002/jnr.22365

[22] Ren TJ, Qiang R, Jiang ZL, Wang GH, Sun L, et al (2013) Improvement in regional CBF by L-serine contributes to its neuroprotective effect in rats after focal cerebral ischemia. PLoS One 8: e67044. 23825613 10.1371/journal.pone.0067044 PMC3692549

[23] de Koning TJ, Snell K, Duran M, Berger R, Poll-the BT, et al (2003) L-Serine in disease and development. Biochem J 371: 653–661. 12534373 10.1042/BJ20021785 PMC1223326

[24] Kramer M, Dang J, Baertling F, Denecke B, Clarner T, et al (2010) TTC staining of damaged brain areas after MCA occlusion in the rat does not constrict quantitative gene and protein analyses. J Neurosci Methods 187: 84–89. 20064557 10.1016/j.jneumeth.2009.12.020

[25] Wang XK, Feuerstein GZ, Xu L, Wang H, Schumacher WA, et al (2004) Inhibition of tumor necrosis factor-α-converting enzyme by a selective antagonist protects brain from focal ischemic injury in rats. Mol Pharmacol 65: 890–896. 15044618 10.1124/mol.65.4.890

[26] Chen J, Li Y, Wang L, Zhang Z, Lu D, et al (2001) Therapeutic benefit of intravenous administration of bone marrow stromal cells after cerebral ischemia in rats. Stroke 32: 1005–1011. 11283404 10.1161/01.str.32.4.1005

[27] Germano AF, Dixon CE, d'Avella D, Hayes RL, Tomasello F (1994) Behavioral deficits following experimental subarachnoid hemorrhage in the rat. J Neurotrauma 11: 345–353. 7996588 10.1089/neu.1994.11.345

[28] Stout RW, Cho DY, Gaunt SD, Taylor HW, Baker DG (2001) Transcutaneous blood gas monitoring in the rat. Comp Med 51: 524–533. 11924815

[29] Joshi CN, Jain SK, Murthy PS (2004) An optimized triphenyltetrazolium chloride method for identification of cerebral infarcts. Brain Res Protoc 13: 11–17. 10.1016/j.brainresprot.2003.12.001 15063836

[30] Manohar R, Appu Rao G, Appaji Rao N (1984) Kinetic mechanism of the interaction of D-cycloserine with serine hydroxymethyltransferase. Biochemistry 23: 4116–4122. 6487593 10.1021/bi00313a016

[31] Ramesh KS, Appaji Rao N (1980) Purification and physicochemical, kinetic and immunological properties of allosteric serine hydroxymethyltransferase from monkey liver. Biochem J 187: 623–636. 6821365 10.1042/bj1870623 PMC1162445

[32] Allen SJ, Watson JJ, Shoemark DK, Barua NU, Patel NK (2013) GDNF, NGF and BDNF as therapeutic options for neurodegeneration. Pharmacol Ther 138: 155–175. 23348013 10.1016/j.pharmthera.2013.01.004

[33] Duarte EP, Curcio M, Canzoniero LM, Duarte CB (2012) Neuroprotection by GDNF in the ischemic brain. Growth Factors 30: 242–257. 22670840 10.3109/08977194.2012.691478

[34] Duan SR, Wang J, Teng W, Xu R (2010) Expression of nestin and glial-derived neurotrophic factor in human endogenous neural stem cells following ischemia. Neurol Res 32: 835–840. 20426902 10.1179/016164109X12581096870159

[35] Chen B, Gao XQ, Yang CX, Tan SK, Sun ZL, et al (2009) Neuroprotective effect of grafting GDNF gene-modified neural stem cells on cerebral ischemia in rats. Brain Res 1284: 1–11. 19520066 10.1016/j.brainres.2009.05.100

[36] Kameda M, Shingo T, Takahashi K, Muraoka K, Kurozumi K, et al (2007) Adult neural stem and progenitor cells modified to secrete GDNF can protect, migrate and integrate after intracerebral transplantation in rats with transient forebrain ischemia. Eur J Neurosci 26: 1462–1478. 17880388 10.1111/j.1460-9568.2007.05776.x

[37] Ichim G, Tauszig-Delamasure S, Mehlen P (2012) Neurotrophins and cell death. Exp Cell Res 318: 1221–1228. 22465479 10.1016/j.yexcr.2012.03.006

[38] Johanson C, Stopa E, Baird A, Sharma H (2011) Traumatic brain injury and recovery mechanisms: peptide modulation of periventricular neurogenic regions by the choroid plexus-CSF nexus. J Neural Transm 118: 115–133. 20936524 10.1007/s00702-010-0498-0 PMC3026679

[39] Sun C, Zhang H, Li J, Huang H, Cheng H, et al (2010) Modulation of the major histocompatibility complex by neural stem cell-derived neurotrophic factors used for regenerative therapy in a rat model of stroke. J Transl Med 8: 77. 20727165 10.1186/1479-5876-8-77 PMC2936305

[40] Schmid RS, Maness PF (2008) L1 and NCAM adhesion molecules as signaling coreceptors in neuronal migration and process outgrowth. Curr Opin Neurobiol 8: 245–250. 10.1016/j.conb.2008.07.015 PMC2633433 18760361

[41] Maness PF, Schachner M (2007) Neural recognition molecules of the immunoglobulin superfamily: signaling transducers of axon guidance and neuronal migration. Nat Neurosci 10: 19–26. 17189949 10.1038/nn1827

[42] Schulz F, Lutz D, Rusche N, Bastús NG, Stieben M, et al (2013) Gold nanoparticles functionalized with a fragment of the neural cell adhesion molecule L1 stimulate L1-mediated functions. Nanoscale 5: 10605–10617. 24056775 10.1039/c3nr02707d

[43] He X, Knepper M, Ding C, Li J, Castro S, et al (2012) Promotion of spinal cord regeneration by neural stem cell-secreted trimerized cell adhesion molecule L1. PLoS One 7: e46223. 23049984 10.1371/journal.pone.0046223 PMC3458024

[44] Cui YF, Xu JC, Hargus G, Jakovcevski I, Schachner M, et al (2011) Embryonic stem cell-derived L1 overexpressing neural aggregates enhance recovery after spinal cord injury in mice. PLoS One 6: e17126. 21445247 10.1371/journal.pone.0017126 PMC3060805

[45] Chen J, Bernreuther C, Dihné M, Schachner M (2005) Cell adhesion molecule L1-transfected embryonic stem cells with enhanced survival support regrowth of corticospinal tract axons in mice after spinal cord injury. J Neurotrauma 22: 896–906. 16083356 10.1089/neu.2005.22.896

[46] Chen J, Joon Lee H, Jakovcevski I, Shah R, Bhagat N, et al (2010) The extracellular matrix glycoprotein tenascin-C is beneficial for spinal cord regeneration. Mol Ther 18: 1769–1777. 20606643 10.1038/mt.2010.133 PMC2951554

[47] Andrews MR, Czvitkovich S, Dassie E, Vogelaar CF, Faissner A, et al (2009) α9 integrin promotes neurite outgrowth on tenascin-C and enhances sensory axon regeneration. J Neurosci 29: 5546–5557. 19403822 10.1523/JNEUROSCI.0759-09.2009 PMC6665849

[48] Meiners S, Mercado ML, Nur-e-Kamal MS, Geller HM (1999) Tenascin-C contains domains that independently regulate neurite outgrowth and neurite guidance. J Neurosci 19: 8443–8453. 10493745 10.1523/JNEUROSCI.19-19-08443.1999 PMC6783022

[49] Joester A, Faissner A (2001) The structure and function of tenascins in the nervous system. Matrix Biol 20: 13–22. 11246000 10.1016/s0945-053x(00)00136-0

[50] Rigato F, Garwood J, Calco V, Heck N, Faivre-Sarrailh C, et al (2002) Tenascin-C promotes neurite outgrowth of embryonic hippocampal neurons through the alternatively spliced fibronectin type III BD domains via activation of the cell adhesion molecule F3/contactin. J Neurosci 22: 6596–6609. 12151539 10.1523/JNEUROSCI.22-15-06596.2002 PMC6758160

[51] Abaskharoun M, Bellemare M, Lau E, Margolis RU (2010) Glypican-1, phosphacan/receptor protein-tyrosine phosphatase-ζ/β and its ligand, tenascin-C, are expressed by neural stem cells and neural cells derived from embryonic stem cells. ASN Neuro 2: e00039. 20689858 10.1042/AN20100001 PMC2914431

[52] Wang T, Xiong JQ, Ren XB, Sun W (2012) The role of Nogo-A in neuroregeneration: a review. Brain Res Bull 87: 499–503. 22414960 10.1016/j.brainresbull.2012.02.011

[53] Pernet V, Schwab ME (2012) The role of Nogo-A in axonal plasticity, regrowth and repair. Cell Tissue Res 349: 97–104. 22588543 10.1007/s00441-012-1432-6

[54] Pernet V, Joly S, Dalkara D, Schwarz O, Christ F, et al (2012) Neuronal Nogo-A upregulation does not contribute to ER stress-associated apoptosis but participates in the regenerative response in the axotomized adult retina. Cell Death Differ 19: 1096–1108. 22193546 10.1038/cdd.2011.191 PMC3374074

